# Spatial-Temporal Kinetic Behaviors of Micron-Nano Dust Adsorption along Epoxy Resin Insulator Surfaces and the Physical Mechanism of Induced Surface Flashover

**DOI:** 10.3390/polym16040485

**Published:** 2024-02-09

**Authors:** Naifan Xue, Bei Li, Yuan Wang, Ning Yang, Ruicheng Yang, Feichen Zhang, Qingmin Li

**Affiliations:** 1 State Key Laboratory of Alternate Electrical Power System with Renewable Energy Sources, North China Electric Power University, Beijing 102206, China; wy_vera0209@163.com (Y.W.); 16608030457@163.com (N.Y.); yrc20000810@163.com (R.Y.); zfc020306@outlook.com (F.Z.); lqmeee@ncepu.edu.cn (Q.L.); 2 School of Electrical Engineering, Hebei University of Technology, Tianjin 300130, China; 2022moltres@gmail.com

**Keywords:** epoxy resin insulator, micron-nano dust, GIS/GIL, adsorption behavior, surface flashover

## Abstract

The advanced Gas Insulated Switchgear/Gas Insulated Lines (GIS/GIL) transmission equipment serves as an essential physical infrastructure for establishing a new energy power system. An analysis spanning nearly a decade on faults arising from extra/ultra-high voltage discharges reveals that over 60% of such faults are attributed to the discharge of metal particles and dust. While existing technical means, such as ultra-high frequency and ultrasonic sensing, exhibit effectiveness in online monitoring of particles larger than sub-millimeter dimensions, the inherent randomness and elusive nature of micron-nano dust pose challenges for effective characterization through current technology. This elusive micron-nano dust, likely concealed as a latent threat, necessitates special attention due to its potential as a “safety killer”. To address the challenges associated with detecting micron-nano dust and comprehending its intricate mechanisms, this paper introduces a micron-nano dust adsorption experimental platform tailored for observation and practical application in GIS/GIL operations. The findings highlight that micron-nano dust’s adsorption state in the electric field predominantly involves agglomerative adsorption along the insulator surface and diffusive adsorption along the direction of the ground electrode. The pivotal factors influencing dust movement include the micron-nano dust’s initial position, mass, material composition, and applied voltage. Further elucidation emphasizes the potential of micron-nano dust as a concealed safety hazard. The study reveals specific physical phenomena during the adsorption process. Agglomerative adsorption results in micron-nano dust speckles forming on the epoxy resin insulator’s surface. With increasing voltage, these speckles undergo an “explosion”, forming an annular dust halo with deepening contours. This phenomenon, distinct from the initial adsorption, is considered a contributing factor to flashovers along the insulator’s surface. The physical mechanism behind flashovers triggered by micron-nano dust is uncovered, highlighting the formation of a localized short circuit area and intense electric field distortion constituted by dust speckles. These findings establish a theoretical foundation and technical support for enhancing the safe operational performance of AC and DC transmission pipelines’ insulation.

## 1. Introduction

The establishment of a new power system integrating multimodal energy sources is an important technical way to realize carbon emission targets, while advanced transmission and transformation equipment is an indispensable physical support for the construction of a new power system. Gas Insulated Switchgear/Gas Insulated Lines (GIS/GIL) are playing crucial roles as the “network joints” and “security guards” of energy transmission [1,2]. GIS/GIL, widely utilized in ultra/high voltage power grids, boasts unique advantages such as minimal transmission loss, substantial capacity, high operational reliability, and integrated transmission of renewable energy sources [3,4,5]. Despite these merits, insulation failure remains the primary cause of GIS/GIL operational issues, with internal metal particle discharge emerging as a key safety concern.

Metal particles are easily moved to the vicinity of the insulators inside the GIS/GIL equipment due to the electric field, making the electric field distortion near the fragile insulator triple junction more serious. Because epoxy resin has good heat resistance and electrical insulation, internally, GIS/GIL mainly use insulators cast from epoxy resin composite materials. Under the electric field, the epoxy resin surface provides an attachment environment for metal particles or micro-nano dust, and the metal contaminants adhering to the epoxy resin surface, or doing the firefly motion near the epoxy resin surface, greatly increase the probability of electrical insulation failure.

For discharge faults triggered by large metal particles (above the submillimeter level), existing UHF and ultrasonic sensing technologies offer acceptable/better online monitoring and early warning capabilities. However, a significant number of unexplained insulation discharge faults persist at engineering sites. Even with current UHF and ultrasonic sensing technologies, better online monitoring and early warning for discharge faults caused by large-size metal particles (sub-millimeter level or larger) are achievable [6,7,8], yet numerous unexplained insulation discharge faults still occur in engineering settings. Through comprehensive analysis, micron-nano dust emerges as a likely “safety killer” with characteristics of high randomness, insidiousness, and inevitability. The smaller size of micron-nano dust, coupled with its strong physical and chemical activity, pronounced randomness in movement, and clustering effects, facilitates its migration near insulators induced by charge dynamics transfer, electromagnetic radiation, acousto-optic radiation, and distinctive statistical characteristics of larger particles [9,10,11]. Unfortunately, existing UHF and ultrasonic sensing technologies prove ineffective in detecting the dispersed motion and discharge signals of nano dust, further hindering the characterization of its electrodynamic properties.

Ruixue Liang [12] investigated the adsorption behavior of micron-sized metal dust, identifying three modes of motion near the insulator: accumulation, diffusion, and others. Notably, the experiment took place in ambient air with PTFE insulator material. While some disparity exists with real-world engineering conditions, Liang’s work lays a foundational understanding for studying micron-scale dust movement characteristics. Jingrui Wang [13] explored the distribution characteristics of metal dust adsorption near insulators with varying inclination angles, revealing that higher angles correlate with reduced dust accumulation. However, limitations arose from simultaneously measuring dust adsorption and flashover breakdown voltage, introducing substantial error, and lowering experimental reliability. In another study, Zhang Lian-gen [14] delved into partial discharge induced by millimeter-level metal foreign matter on insulator surfaces, describing the persistent erosion caused by dense micro-discharge patterns under constant voltage. Yet, the adsorption and discharge mechanisms of micrometer-level metal foreign matter were left unexplored. Xu Yuan [15] summarized the motion characteristics of different quantities of 100-micron metal particles on GIS insulator surfaces, qualitatively analyzing correlations with partial discharge characteristics but without establishing direct links.

Considering the studies, while it is recognized that fine metal dust in GIS/GIL readily adsorbs onto insulator surfaces, the kinetic behavior and adsorption mechanism remain unclear. Building on existing research on the motion behavior and discharge characteristics of millimeter-sized metal particles, this paper establishes a metal powder adsorption experimental platform suitable for GIS/GIL observation and operation. The focus is on the impact of aberrant electric fields and microscopic force fields on metal dust kinetic behavior patterns. The investigation aims to develop a multi-physical force analysis model for metal dust, unraveling the adsorption mechanism near GIS/GIL basin insulators. This research provides a fundamental basis for dust protection and insulation design.

## 2. Experimental Platform and Parameter Setting

**Figure 1:**
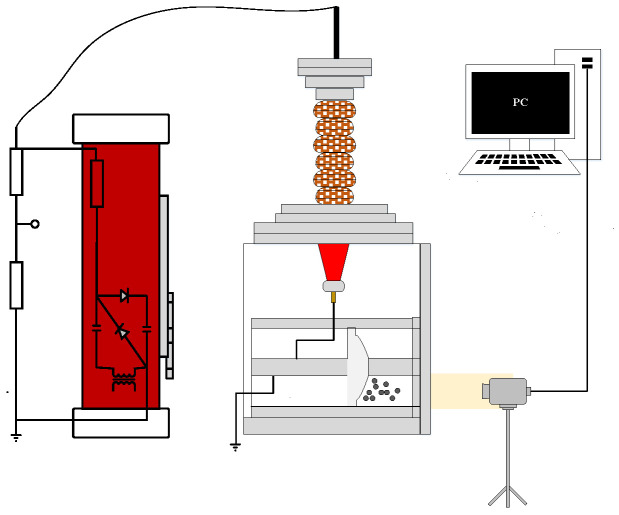
Schematic diagram of the experimental platform for coaxial cylindrical electrodes.

**Figure 2:**
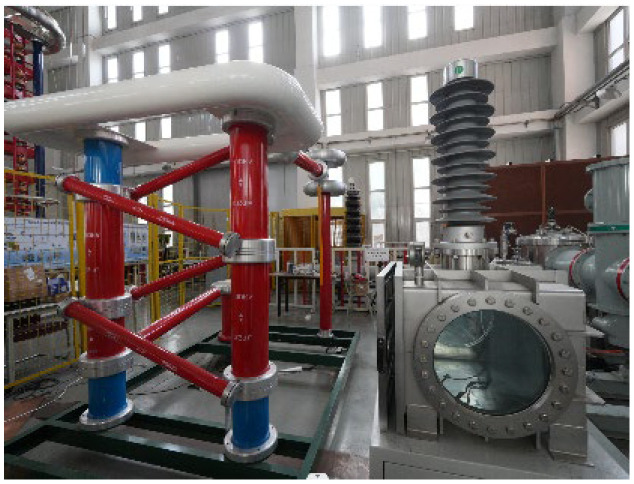
True type power supply and experimental chamber.

To elucidate the adsorption dynamics of micronized dust along the gas-solid interface of insulators in GIS/GIL, we devised a schematic diagram depicting the coaxial cylindrical electrode experimental platform, as illustrated in Figure 1. The experimental setup comprises three main components: the experimental chamber housing the coaxial cylindrical model, the DC power source, and the data processing unit. The actual DC power supply and experimental chamber configuration are detailed in Figure 2.

**Figure 3:**
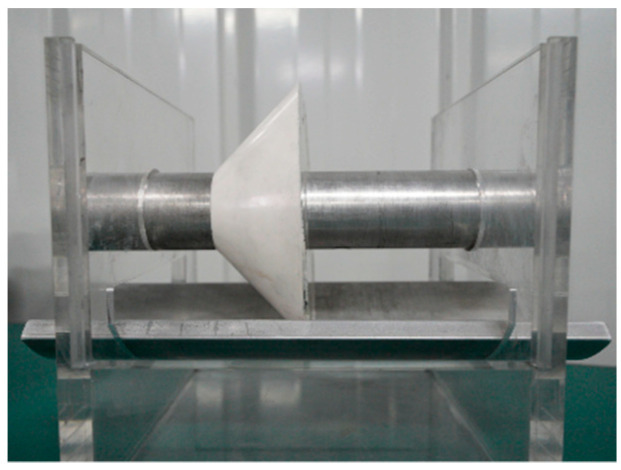
Scaling model of a coaxial cylindrical electrode.

**Figure 4:**
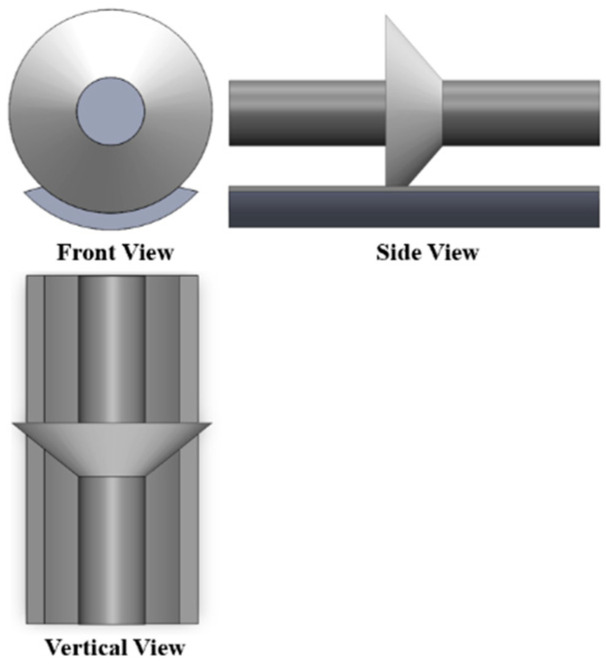
Three views of a coaxial cylindrical electrode.

The coaxial cylindrical model, depicted in Figure 3, is a scaled representation comprising a high-voltage electrode and a ground electrode. The high-voltage electrode is an aluminum cylinder measuring 40 mm in diameter and 260 mm in length, while the ground electrode is a cylindrical aluminum shell with an inner diameter of 120 mm, an outer diameter of 140 mm, and a length of 260 mm. Both electrodes are supported by a transparent acrylic plate, ensuring their center axes coincide. The coaxial cylindrical electrode platform employs a scaled basin insulator featuring an inner diameter of 40 mm, an outer diameter of 120 mm, a thickness of 40 mm, and an inclination angle of 45 degrees. The chemical composition of the scaled basin insulator used in the experiment includes epoxy resin, alumina, and a curing agent. Bisphenol A diglycidyl ether (DGEBA) was used as the epoxy monomer, and methyl tetrahydrophthalic anhydride (MTHPA) was used as the curing agent. The mass ratio between the components was epoxy resin 80:alumina 100:curing agent 330. The curing condition was as follows: cure at 100 °C for 4 h, then increase the temperature, and then continue to cure at 150 °C for 10 h. Alumina is the main material to improve the performance of epoxy resin because of its high mechanical strength and strong chemical stability. Adding alumina can effectively improve the impact strength, bending strength, thermal conductivity, and glass transition temperature of epoxy insulating materials, and to a certain extent, increase the resistivity and improve the flashover resistance. Moreover, the alumina material could resist the corrosion of sulfur hexafluoride decomposition gas. Figure 4 provides three perspectives of the coaxial cylindrical scaled model.

The high-voltage electrode connects to the DC power supply via a high-voltage wire within the experimental chamber, while the ground electrode links to the ground wire, establishing a comprehensive electrical circuit. Throughout the experiment, the coaxial cylindrical electrode is positioned within a square closed experimental chamber, featuring a transparent observation window on one side. A supplementary light source enhances the overall chamber illumination, and a high-definition camera, positioned on the opposing side, records the dust adsorption process.

**Figure 5:**
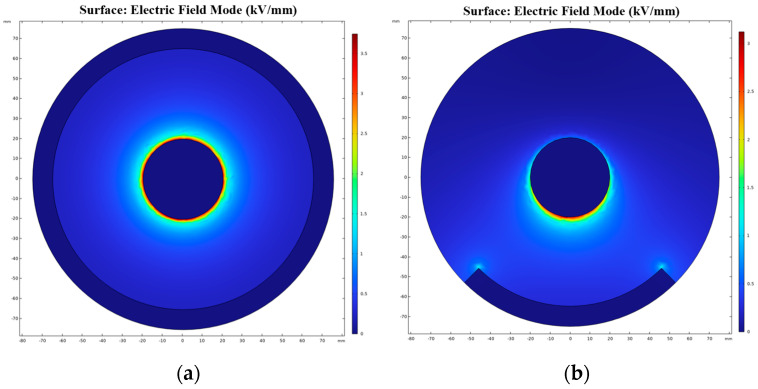
E-field distribution of the test rigs. (**a**) Fully enclosed chamber; (**b**) Semi-closed chamber.

To validate the equivalence of the electric field in the semi-enclosed coaxial cylindrical experimental platform and the fully enclosed coaxial cylindrical real platform of GIS/GIL, finite element simulation is employed. Simulation models for semi-enclosed and fully enclosed coaxial columns are developed, with the electric field distribution at the gas-solid interface obtained under a 25 kV voltage in an SF6 gas environment. Given the placement of dust at the bottom of the insulator’s convex side during the experiment, the dust adsorption area aligns roughly within 45 degrees of the gas-solid interface corresponding to the ground electrode. The simulation results in Figure 5 indicate that the maximum field strength in Figure 5a is 3.74 kV/mm, and in Figure 5b it is 3.12 kV/mm, with the size of the field distribution being essentially identical. The maximum field strength near the surface of the epoxy resin in a 126 kV true GIS/GIL is around 3.5 kV/mm. This confirms that the coaxial cylindrical electrodes employed in the experiment are equivalent to the SF6 gas simulation platform, affirming the effectiveness and validity of the experimental setup.

**Figure 6:**
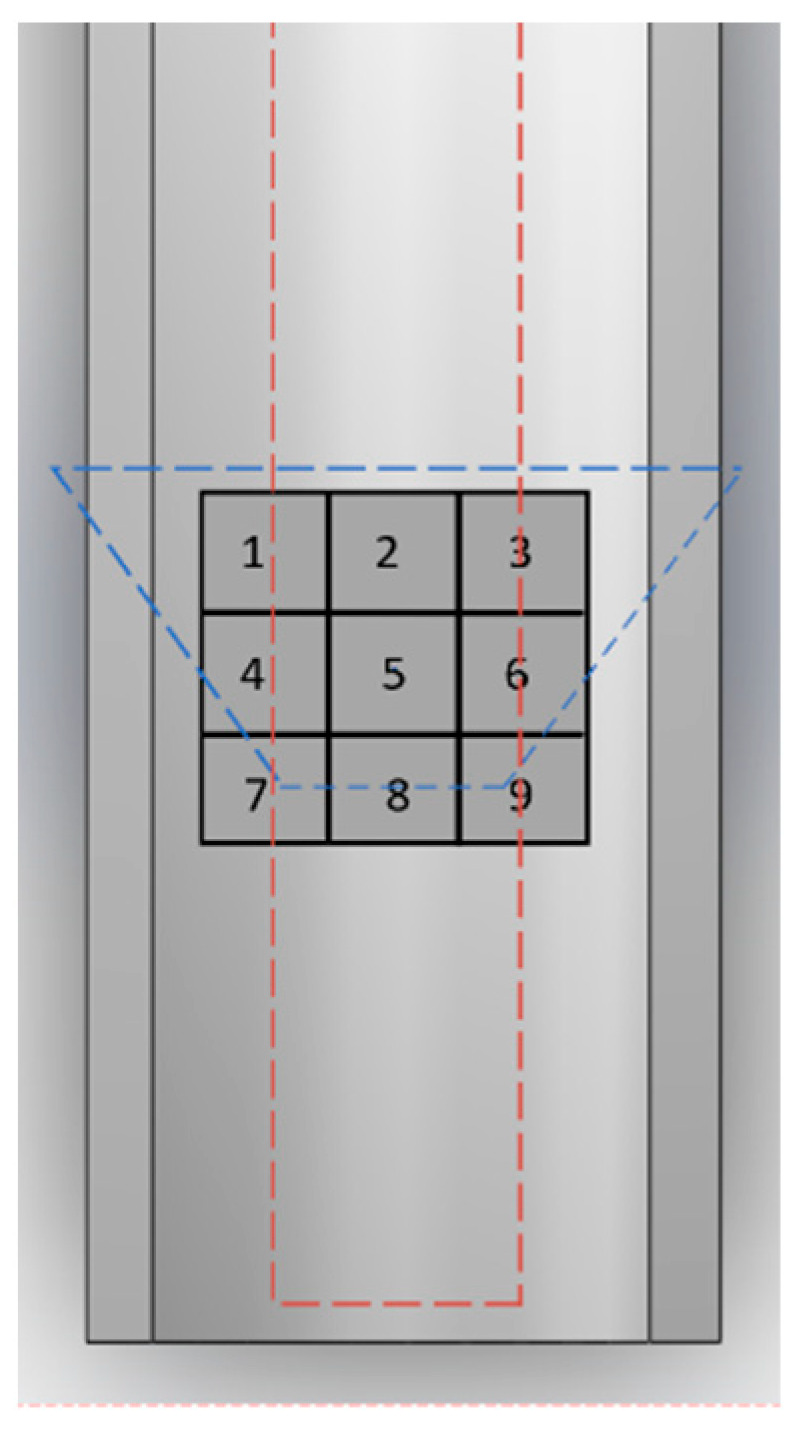
Initial positions of dust.

The experimental setup positioned the dust, as depicted in Figure 6, on top of the convex insulator’s ground electrode. Figure 6 is the vertical view in Figure 4. The blue dotted line represents the top view profile of the insulator, and the red dotted line represents the top view profile of the high-voltage electrode. The placement was segmented into a nine-grid configuration, with combinations in positions 1–3 and 7–9 representing the furthest distances among the three combinations. Before initiating the experiment, aluminum powder underwent more than 24 h of drying in an oven to ensure complete dryness, mitigating the potential impact of moisture on experimental outcomes. To minimize the influence of prior experiments on subsequent iterations, a thorough wipe-down of the high-voltage electrode, ground electrode, insulator surface, and internal cavity with ethanol preceded each new round of experiments. This step aimed to eliminate surface charge effects, with experiments commencing after the ethanol had evaporated. Precise control was maintained using a high-precision balance (with an error not exceeding 0.1%) to measure the required dust quantity. The experiments were conducted at a temperature of 25 °C, within a 0.1 MPa SF6 environment, with a boosting speed of 2 kV/s. Each set of experiments underwent five repetitions to calculate an average value, thus reducing uncertainties arising from accidental phenomena in the experimental results.

The typical forces of micron-nano dust are shown in Table 1, including gravity G, buoyancy F_b_, electrical force F_E_, coulomb force F_q_, van der Waals force between two contiguous dusts F_vdW_, van der Waals force between two untouched dusts F, van der Waals force between dust and surface F_Rumpf_, and adhesive force F_adhe_.

## 3. Kinetic Behavior of Micron-Nano Dust Adsorption

### 3.1. General Adsorption Behavior of Micron-Nano Dust

**Figure 7:**
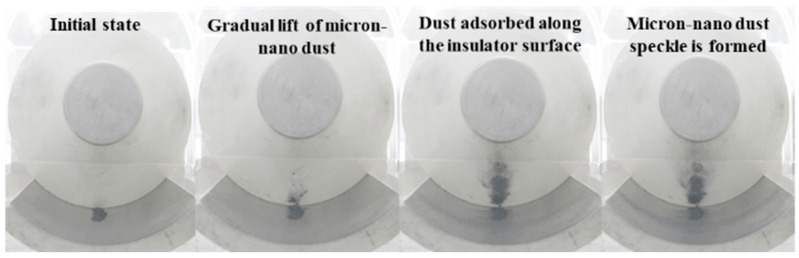
Adsorption behavior of micron-nano aluminum dust parallel to a high-voltage conductor (position 258).

**Figure 8:**
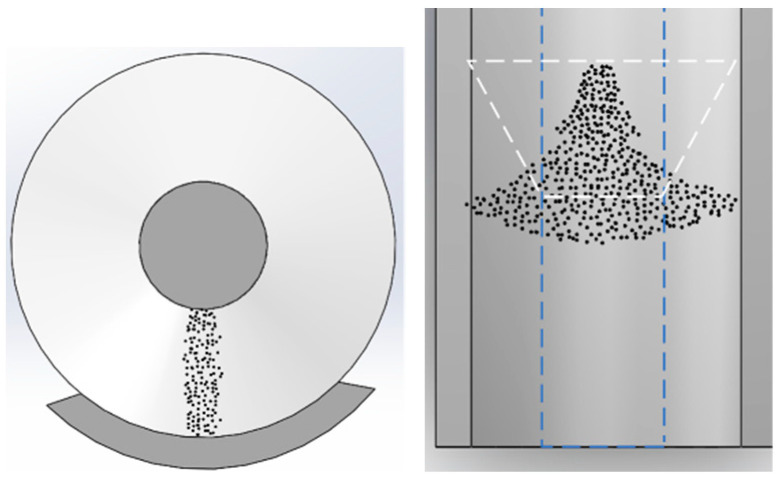
Agglomerative adsorption along the insulator surface, diffusive adsorption in the direction of the ground electrode.

A uniform deposition of 20 mg of 50 nm aluminum dust occurs on the ground electrode, aligned parallel to the high-voltage conductor at position 258. A negative polarity DC voltage is applied, following the procedures outlined in the initial section, to observe the real-time adsorption movement behavior of micron-nano dust along the insulator surface as voltage increases. The position of the insulator housing the dust is captured in Figure 7. Notably, the 50 nm aluminum dust appears silver-black due to optical dispersion effects. Experimental findings indicate that micron-nano dust gradually begins to lift from −10 kV. In contrast to larger particles, the lifting of micron-nano dust is a continuous process, with not all particles lifting simultaneously. Therefore, this paper focuses on recording the starting lifting voltage of micron-nano dust. As the voltage increases progressively, the movement of micron-nano dust in the electric field primarily manifests in two forms: agglomerative adsorption along the insulator interface and diffusive adsorption along the direction of the ground electrode, as depicted in Figure 8.

Upon reaching voltages beyond −10 kV, micron-nano dust undergoes elevation and migrates towards the insulator surface. With a continuous increase in voltage, the adsorption quantity of micron-nano dust progressively rises, leading to darker and more substantial agglomerations of micron-nano dust. At −40 kV, agglomerated micron-nano dust forms distinctive patches and lumps on the convex insulator surface, herein referred to as micron-nano dust speckles. These speckles exhibit stable adhesion to the insulator surface, with the adsorption state and quantity remaining relatively constant if the voltage increase is halted. However, if voltage escalation persists, existing micron-nano dust speckles at the gas-solid interface emerge as pivotal factors inducing subsequent flashovers along the surface of the epoxy resin insulator.

Regarding the diffusion and adsorption behavior of micron-nano dust along the direction of the ground electrode, when the voltage surpasses −15 kV, both the diffusion and adsorption area of micron-nano dust expand, accompanied by an increase in diffusion and adsorption quantity. A penetrating dust band forms perpendicular to the direction of the high-voltage conductor on the ground electrode’s surface. The color of this dust band intensifies with the gradual voltage increase, resembling a “tree-like” structure in a top-view perspective. It is noteworthy that, considering solely the movement of dust adsorption, after the formation of micron-nano dust patches and the completion of diffusive adsorption on the ground electrode, a surplus of the initial 20 mg of micron-nano dust persists. Subsequent experiments during this period do not observe the phenomenon of complete adsorption of the initial dust. This remaining pile of micron-nano dust provides ample metal reactants for subsequent discharge chemical reactions triggered by flashovers along the surface.

### 3.2. Key Factors Affecting the Behavior of Micron-Nano Dust Motions

Experiments involving the adsorption of 20 mg of 50 nm aluminum dust reveal a direct correlation between the initial state of the dust and its subsequent adsorption behavior along the epoxy resin insulator. This paper systematically identifies and summarizes four key factors influencing the adsorption behavior of micron-nano dust: the initial position of the dust, its initial mass, the type of electric current (AC/DC), and the material composition of the dust.

#### 3.2.1. Adsorption Behavior of Micronized Dust at Different Initial Positions

**Figure 9:**
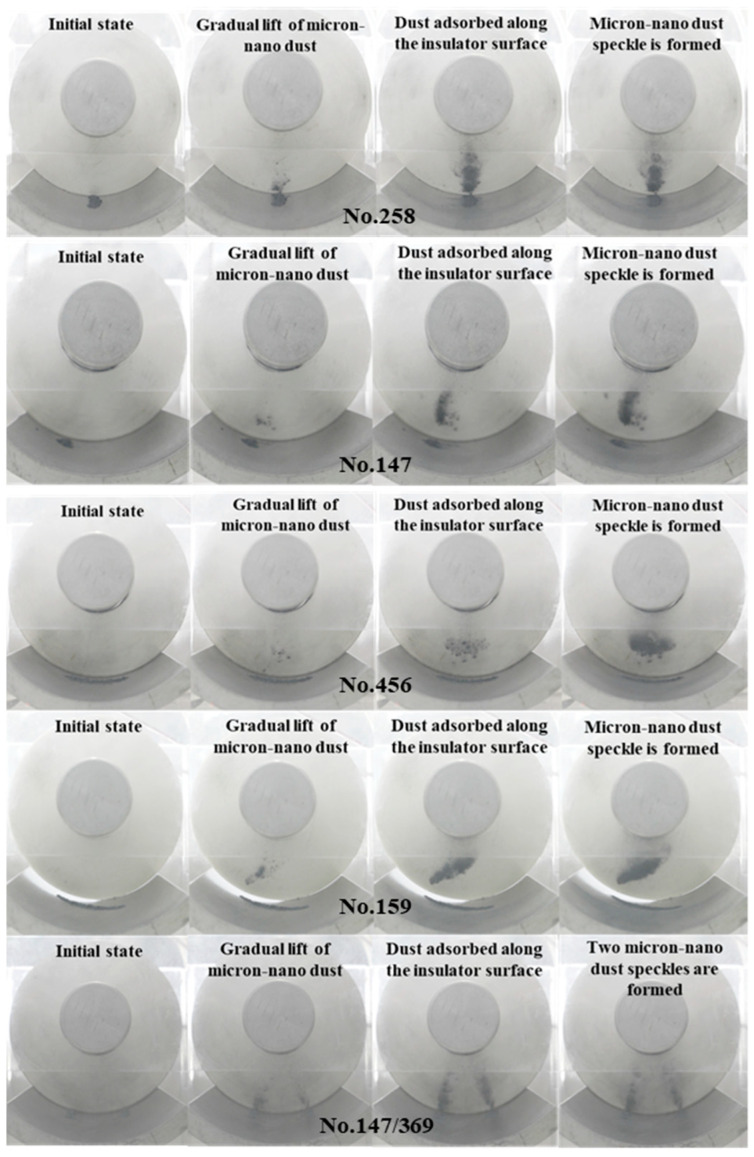
Micron-nano dust adsorption behaviors under different initial circumstances.

In the experiment depicted in Figure 7, micron-nano dust is arranged parallel to the high-voltage conductor. At position 258, the micron-nano dust is positioned directly below the high-voltage conductor. Using this position as a reference point, adsorption experiments are conducted with 20 mg of 50 nm aluminum dust along the surface of the epoxy resin insulator. The dust is arranged at position 147, parallel to the high-voltage conductor and below the side, at position 456, perpendicular to the direction of the high-voltage conductor, at position 159, across the arrangement area, and at the dual initial position 147/369, parallel to the high-voltage conductor. The dynamic aspects of the adsorption behavior are illustrated in Figure 9.

The complete adsorption processes of micron-nano dust at the initial positions of 147, 456, 159, and 147/369 closely mirror that of the dust at position 258, as depicted in Figure 9. The 50 nm aluminum dust gradually lifts from −12 kV, −10 kV, −15 kV, and −10 kV, adhering radially upward along the coaxial cylindrical electrodes, exhibiting an upward-rightward slanting adsorption state from the main view. As the voltage progressively increases, the quantity of inclined agglomerated micron-nano dust adsorption rises, accompanied by a deepening color. By the time the voltage reaches −40 kV, −42 kV, −42 kV, and −44 kV, stable tilted micron-nano dust speckles form on the surface of the epoxy insulator. Concerning the diffuse adsorption behavior on the ground electrode, the overall development process mirrors that of the dust at position 258. Taking the experiment based on position 147 as an example, when the voltage surpasses −18 kV, the dust simultaneously disperses along the ground electrode, away from the insulator’s direction and in the direction of the vertical high-voltage conductor. Since the initial position is on the left side of the ground electrode, the resulting diffusion traces exhibit distinct left-deep-right-shallow characteristics.

Analysis reveals that the adsorption behaviors of the two types of micron-nano dust, agglomerated and diffused along the ground electrode arranged parallel to the high-voltage conductor (positions 258 and 147), are similar. The deviation in the corresponding voltage values of the dynamic process is relatively small. This proximity arises from the modest difference in the required climb height for the lifting of 50 nm aluminum dust at the two initial positions, thus leading to closely aligned voltage values essential for the formation of analogous adsorption characteristics. In contrast to the initial two sets of experiments, the length of the transverse micron-nano dust patches along the radial direction of the insulator is notably shorter. This variation presents the possibility of subsequently impeding the development of flashovers along the insulator surface. Although the deviation in voltage values corresponding to the dynamic adsorption process is minimal, it is closely aligned with that of the formation of dust patches on parallel high-voltage conductors. Due to the extensive distribution of micron-nano dust arranged according to an initial position on the surface of the ground electrode (position 159), the time and voltage required from the start of lifting to the stable micron-nano dust patch formation are higher, by 25%, than that of the equivalent mass of dust starting at position 258.

Figure 9 also illustrates the adsorption process of micron-nano dust at dual initial positions, specifically chosen parallel to the high-voltage conductor at positions 147 and 369. It is noteworthy that the total mass of the two piles of micron-nano dust remains at 20 mg. In comparison with the single micron-nano dust speckle formed by agglomerative adsorption at position 147, the double micron-nano dust speckle formed is noticeably lighter in color and smaller in adsorption area.

While the process of dust speckle formation for different initial positions of micron-nano dust exhibited minimal differences, there was a significant variation in the final morphology of the formed micron-nano dust speckle, as well as the time and voltage required. Therefore, the initial position of micron-nano dust emerges as a crucial factor influencing the morphology of micron-nano dust speckles formed through agglomerative adsorption.

#### 3.2.2. Adsorption Behavior of Micron-Nano Dust with Different Initial Masses

From the above analysis of the double micron-nano dust speckles shown in Figure 9, it is evident that the double micron-nano dust speckle is notably lighter in color and has a smaller adsorption area compared to the single micron-nano dust speckle formed by agglomerative adsorption at location 147. This difference arises because the double initial dust pile is smaller in mass than the single pile, and the total amount utilized for agglomerative adsorption is less than that of the single dust pile.

**Figure 10:**
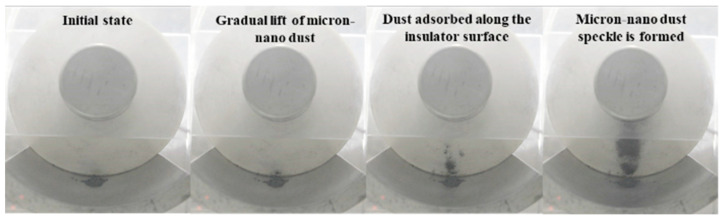
Adsorption behavior of 40 mg of micronized dust at position 258.

Figure 10 illustrates the adsorption process of 40 mg of 50 nm micron-nano aluminum dust with the initial position located in the direction of the parallel high-voltage conductor (position 258). The dust gradually lifts from −10 kV, and the agglomerative adsorption process does not significantly differ from the dynamic adsorption process shown in Figure 7. The dust speckle is also formed at −40 kV, but the surface area of the micron-nano dust speckle formed by the 40 mg dust is larger, and the color is darker. Regarding the diffusive adsorption behavior of micron-nano dust along the direction of the ground electrode, the higher the mass, the larger the diffusive adsorption area. Due to the limited size of the ground electrode used in the experiments, most of the dust diffused to the area outside the ground electrode.

**Figure 11:**
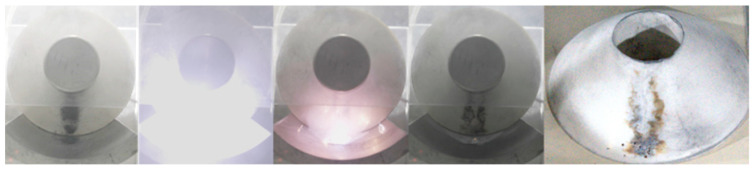
Micron-nano dust speckle induced a flashover along the surface when the initial dust mass was greater than or equal to 40 mg.

Simultaneously, experiments conducted with varying initial masses at the same position reveal a more pronounced agglomerative adsorption phenomenon with an increase in the initial mass. For micron-nano dust positioned at location 258, when its initial mass is 40 mg or greater, the insulator surface experiences an intense along-face flashover phenomenon shortly after the formation of a micron-nano dust speckle, as depicted in Figure 11. Considering that the micron-nano dust speckle induces a flashover breakdown voltage of −40 kV along the surface, equivalent to the air breakdown voltage of the test system, it can be postulated that micron-nano dust with an initial mass greater than or equal to 40 mg, situated in position 258, will lead to a 0.1 MPa SF6 failure.

Based on the experimental phenomena and analysis, it is apparent that the dynamic process of dust patch formation remains fundamentally consistent across different initial masses of micron-nano dust. However, there is a substantial disparity in the final formation of micron-nano dust patches in terms of surface area, and when the mass surpasses a critical threshold, these dust patches significantly diminish the insulation margin of the gas-solid interface, directly leading to flashovers along the surface. Consequently, the initial mass emerges as a crucial factor influencing the behavior of micron-nano dust movement.

#### 3.2.3. Adsorption Behavior of Micronized Dust When Externally Applied Voltage Is AC

**Figure 12:**
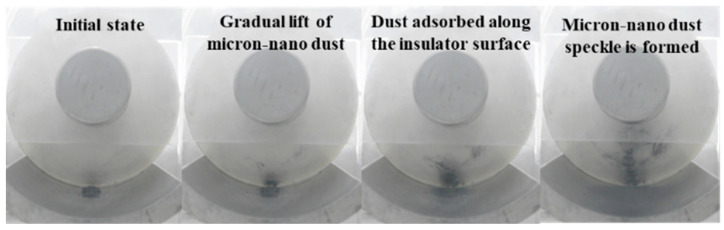
Adsorption behavior 20 mg of micron-nano dust at position 258 under AC voltage.

Figure 12 illustrates the adsorption process of a 20 mg, 50 nm micron-nano aluminum speckle under externally applied AC voltage, with the initial position situated in the direction of the parallel high-voltage conductor (position 258). The micron-nano dust initiates at a voltage of 10 kV, and the corresponding voltage for the formation of the micron-nano dust speckle is 42 kV. It is noteworthy that the stability of the micron-nano dust speckle formed under AC voltage is inferior to that under DC voltage. Concurrently, the diffusion and adsorption behavior of the ground electrode differs significantly. High-definition camera observations reveal that the micron-nano dust generates an “air wave” on the surface of the ground electrode, oscillating back and forth with the alternating electric field in the direction of the parallel high-voltage conductor.

#### 3.2.4. Adsorption Behavior of Micron-Nano Dust from Different Materials

**Figure 13:**
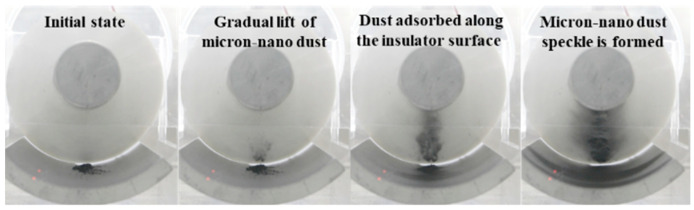
Adsorption behavior of micron-nano copper dust at a parallel high-voltage conductor (position 258).

The adsorption experiments involved the placement of 100 mg of 50 nm copper dust at position 258 in the direction of the parallel high-voltage conductor, as depicted in Figure 13. The behavior of the 50 nm copper dust is observed as it gradually lifts from −14 kV and adsorbs radially upward along the coaxial cylindrical electrode. With the incremental increase in voltage, there is a gradual augmentation in the amount of tilted, agglomerated micron-nano dust adsorption, accompanied by a deepening color. By the time the voltage reaches 40 kV, stable, tilted micron-nano dust spots manifest on the surface of the epoxy insulator. Regarding the diffuse adsorption behavior on the ground electrode, the overall developmental process mirrors that of the dust at position 258. Once the voltage surpasses −10 kV, the dust spreads concurrently along the ground electrode, extending away from the insulator and perpendicular to the high-voltage conductor. This results in a ground electrode diffusion trace that is notably deeper than the trace formed by the aluminum dust.

In summary, as the voltage steadily rises, the adsorption state of micron-nano dust in the electric field primarily involves agglomerative adsorption along the insulator surface and diffusive adsorption along the direction of the ground electrode. The pivotal factors influencing the motion behavior encompass the initial position of the micron-nano dust, its mass, material composition, and the externally applied voltage.

## 4. Dynamic Processes Prior to Flashover along the Surface Induced by Attached Micron-Nano Dusts

To investigate the physical mechanism of micron-nano dust-induced flashover along the gas-solid interface of epoxy resin insulators, we extended our observations from the micron-nano dust adsorption behavior experiments mentioned above. We systematically increased the voltage to examine the flashover induced by the micron-nano dust speckle formed along the surface.

### 4.1. Special Physical Phenomena Prior to Flashover along the Surface

**Figure 14:**
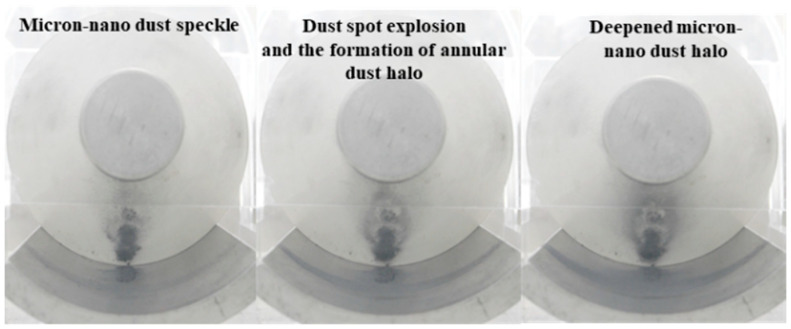
Special physical phenomena of 20 mg 50 nm aluminum dust speckle at position 258.

As depicted in Figure 7, during the adsorption experiment with 20 mg of 50 nm aluminum dust starting at position 258, stable micron-nano dust speckles formed on the epoxy insulator’s surface when the voltage reached −40 kV. As we further increased the voltage, a unique physical phenomenon, distinct from the initial lift adsorption, emerged, as illustrated in Figure 14.

Continuing voltage augmentation revealed that the phenomenon of agglomerative adsorption of micron-nano dust became less prominent, and the micron-nano dust speckle remained stably adsorbed on the insulator surface. At −52 kV, an intriguing event occurred: the micron-nano dust speckle abruptly “exploded”, creating a ring-shaped micron-nano dust halo above the speckle. This ring-shaped halo, similar to the dust speckle, would stably adhere to the epoxy resin insulator’s surface if the voltage was not increased further. Notably, the “explosion” of the dust speckle to form the dust halo was a brief event, occurring immediately after reaching −52 kV and concluding rapidly. This “explosion” lacked components of a redox reaction, in a chemical sense, but represented a physical phenomenon of rapid expansion. Concurrently, the ground electrode diffusion adsorption phenomenon intensified, enlarging the diffusion area, and deepening in color.

Following the formation of the ring-shaped micron-nano dust halo, further voltage escalation resulted in a notable instantaneous adsorption of the residual micron-nano dust on the ground electrode. Most of these particles adhered near the recently formed ring-shaped micron-nano dust halo, leading to a significant deepening of the halo, as evident in Figure 14. Simultaneously, rapid diffusion occurred among the micron-nano dust on the ground electrode.

#### 4.1.1. Formation and Contour Deepening of Micron-Nano Dust Halos at Different Initial Positions

**Figure 15:**
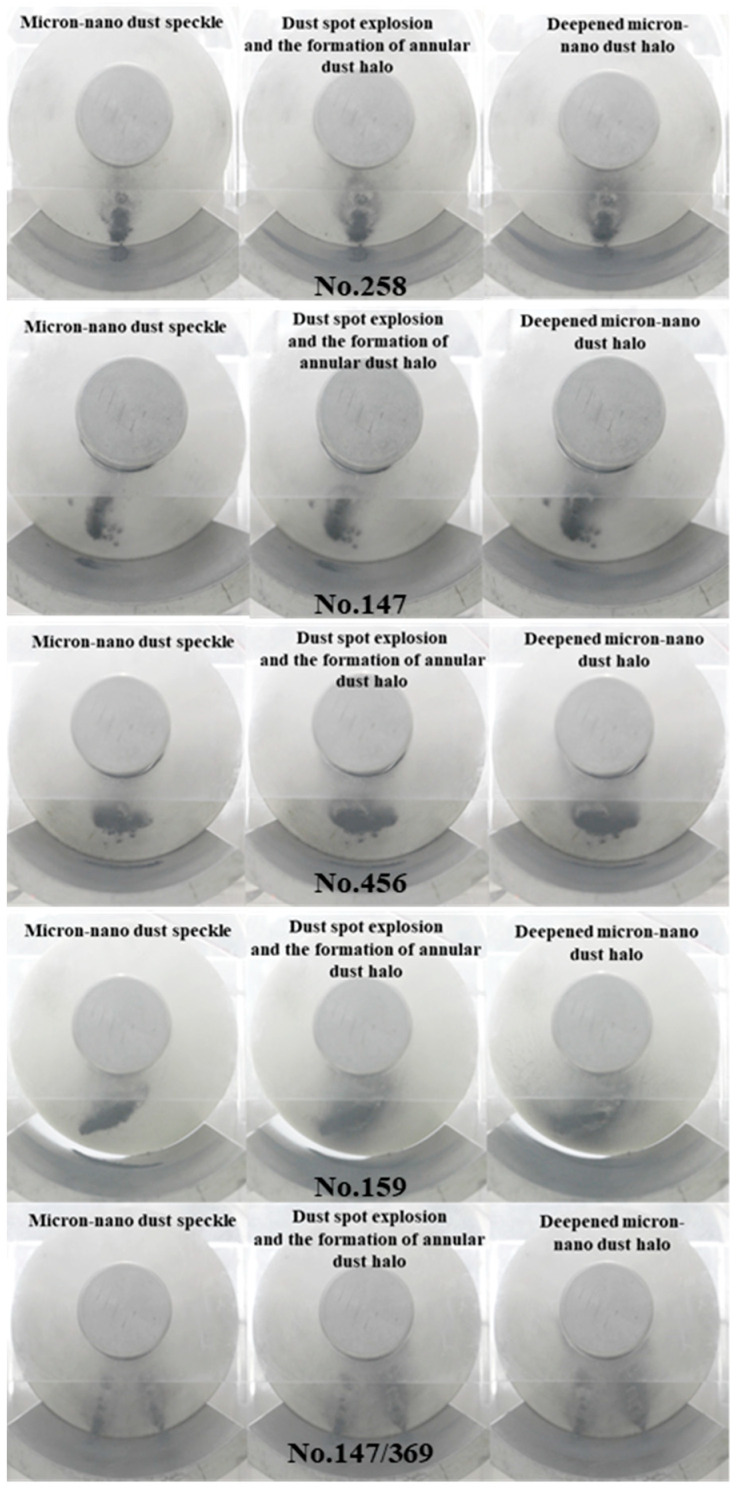
Special physical phenomena of dust speckles under different circumstances.

Illustrated in Figure 9, the adsorption experiments involving 20 mg of 50 nm aluminum dust at the initial positions 147, 456, 159, and 147/369 exhibit the formation of a stable micron-nano dust speckle on the epoxy insulator surface when the voltage reaches −40 kV, −42 kV, −42 kV, and −44 kV. Upon further voltage increase to −60 kV, −52 kV, −60 kV, and −60 kV, the micron-nano dust speckle undergoes an “explosion”, resulting in the creation of a ring-shaped micron-nano dust halo diagonally above the dust speckle. At −66 kV, −64 kV, −66 kV, and −70 kV, a noticeable one-time instantaneous adsorption occurs among the residual micron-nano dust on the ground electrode. The majority of these particles adhere to the vicinity of the recently formed annular micron-nano dust halo, significantly deepening it, as depicted in Figure 15.

Despite variations in the morphology of the micron-nano dust speckles formed at distinct initial positions, the experimental phenomena involving the explosion formation of annular dust halos and the deepening of dust halo contours are consistently observed under DC voltage as the voltage increases.

#### 4.1.2. Formation and Contour Deepening of Micron-Nano Copper Dust Halos

**Figure 16:**
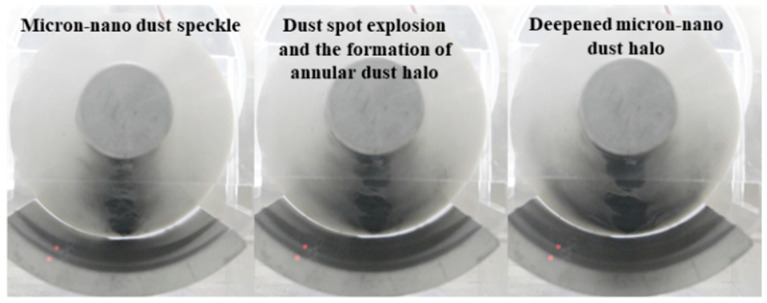
Copper dust halo formation and contour deepening at position 258.

As depicted in Figure 16, in the adsorption experiment conducted with copper dust starting at position 258, when the voltage reaches −40 kV, a micron-nano copper dust speckle forms on the surface of the epoxy resin insulator. Upon further voltage increase to −60 kV, the micron-nano dust speckle undergoes an “explosion”, resulting in the formation of dust halos on both sides of the speckle. Although not forming a closed circle, the dust halo exhibits an arc distribution, contributing to the creation of a ring-shaped micron-nano dust halo. At −72 kV, the contour of the dust halo deepens, as depicted in Figure 16.

#### 4.1.3. Special Physical Phenomena Prior to Flashover along the Surface under AC Voltage

**Figure 17:**
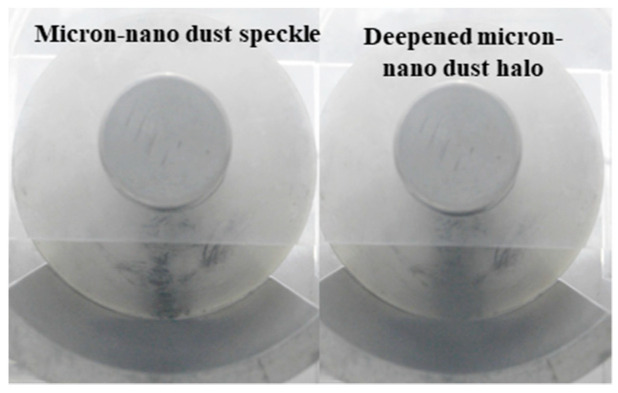
20 mg, 50 nm aluminum dust band at position 258 under AC voltage.

As illustrated in Figure 15, during the adsorption experiment involving 20 mg of 50 nm aluminum dust at the initial position 258 under externally applied AC voltage, upon reaching −42 kV, an unstable dust speckle agglomerates on the surface of the epoxy resin insulator. Subsequently, at 52 kV, the micron-nano dust speckle undergoes an “explosion”, resulting in the formation of a larger surface area of micron-nano dust band, as shown in Figure 17. In contrast to DC voltage, AC voltage does not lead to the formation of a dust halo during the dust speckle explosion; instead, it directly completes an instantaneous adsorption, forming a larger area of agglomeration in the band dust speckle.

In summary, when different materials of micron-nano dust are subjected to the same applied voltage, the experimental phenomenon of dust speckle explosion leading to the formation of a ring-shaped dust halo, followed by the deepening of the dust halo contour, varies. Copper dust and aluminum dust form similar dust speckles, but the shape of the dust halo resulting from the dust speckle “explosion” exhibits different morphologies, including arcs and rings. Nevertheless, it is undeniable that, as the voltage applied to the micron-nano dust speckle continues to rise, it gives rise to special physical phenomena distinct from the initial adsorption, thus creating necessary conditions for induced flashover along the surface.

### 4.2. Dominant Factors Contributing to the Occurrence of Dust Spot Explosions

In the pressurization process, the presence of stable charge micron-nano dust speckles on the surface of the epoxy resin insulator induces severe distortions in the gas-solid interface electric field. Dust particles experience intense electric field distortions within the gap of the dust speckle, leading to SF6 gas partial discharge. As a consequence, dust adheres to the insulator surface, causing an increase in internal temperature and promoting the agglomeration of dust particles within the speckle. This agglomeration results in a considerable amount of energy, causing excessive stress inside the dust speckle. The heightened stress leads to the outward splashing of dust particles, presenting a macroscopic “explosion phenomenon”.

Simultaneously, micron-nano dust particles initially positioned on the ground electrode acquire the same charge induced by the high-voltage conductor. With the rising voltage, these similarly charged micron-nano dust particles lift to the insulator surface, forming micron-nano dust speckles. As the voltage continues to increase, Coulomb repulsion among the like-charged micron-nano dust particles within the speckle gradually intensifies. The lifting of dust and subsequent collisions disturb the equilibrium of the dust speckle, causing a small number of dust particles to bounce outward. This process is also characterized by a macroscopic “explosion phenomenon”.

## 5. Physical Mechanism of Interfacial Adsorption of Micron-Nano Dust-Induced Flashover along the Epoxy Resin Surface

**Figure 18:**
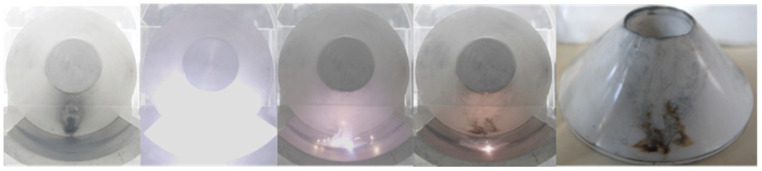
Surface flashover induced by 20 mg of 50 nm aluminum powder at position 258.

As depicted in Figure 15, the contour of the micron-nano dust halo experiences a pronounced deepening at −60 kV. With the ongoing increase in voltage, at −64 kV, the agglomerated micron-nano dust adhered to the surface of the epoxy resin insulator induces surface flashover. This flashover is characterized by the violent ablation of the epoxy resin surface. Furthermore, the current flowing along the surface during the flashover initiates a direct and intense reaction between the residual metal dust on the ground electrode and SF6, as illustrated in Figure 18.

### 5.1. Interfacial Adsorption of Micron-Nano Dust-Induced Flashover along the Surface

**Figure 19:**
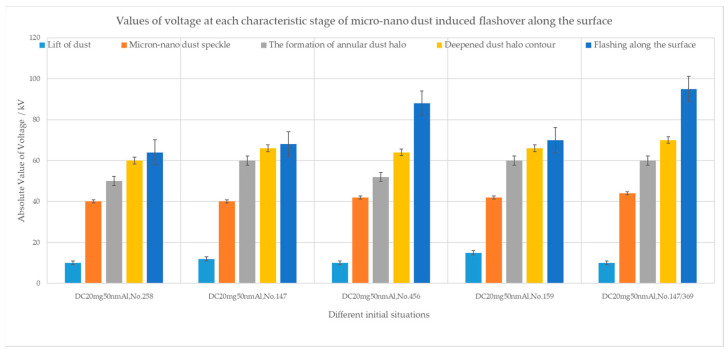
Values for the characteristic stages of micron-nano dust adsorption.

Despite differences in micron-nano dust speckle formation, the emergence of ring-shaped dust halo explosions, and the deepening of the dust halo contour, the flashover development consistently follows a path through the micron-nano dust speckle. The flashover current traverses the dust halo and dust speckle, sufficiently reacting at the epoxy resin surface to ultimately ignite residual dust on the ground electrode. In Figure 19, voltage values for each characteristic stage of micron-nano dust adsorption along the insulator surface leading to surface flashover are presented under diverse initial conditions.

Upon analysis of Table 1, it becomes evident that the requisite condition for micron-nano dust to induce surface flashover under DC voltage is the formation of a micron-nano dust speckle and the subsequent “explosion” of the dust speckle. Unlike large-sized particles, DC voltage is more likely to cause insulation failure due to its unidirectional force, whereas the opposite holds true for micron-nano dust. Micron-nano dust poses a greater hazard under AC voltages, exhibiting significantly lower breakdown voltages than in DC environments under identical conditions.

Under DC voltage, the flashover voltage along the surface is greatly influenced by the initial position, initial mass, and dust material, with micron-nano dust reducing the breakdown voltage of the test system by up to 71%.

### 5.2. Mechanism of Action for Inducing Flashovers along the Surface

Micron-nano dust exhibits pronounced conductivity, and the agglomeration adsorption forming micron-nano dust speckles on the surface of epoxy resin insulators establishes a localized short-circuit region. This short-circuit region reduces the insulation distance between the high-voltage conductor and the grounding shell, making flashover more likely as the length of the dust speckle along the insulator bus direction increases, elongating the “short-circuit region” between the high and low voltage electrodes.

Microscopic analysis reveals that agglomeration adsorption along the epoxy resin surface of micron-nano dust spots leads to varying forms of existence and arrangements, causing different degrees of distortion in the surrounding electric field. In the discharge process, aluminum and copper micron-nano dust primarily exist in clusters, resulting in the most significant electric field distortion at the center sphere, reaching up to 4.5 times the original field strength. However, dust generated in the GIS/GIL cavity typically assumes irregular geometries due to mechanical wear, with dust having multiple spikes, leading to more severe local electric field distortion.

This distorted electric field exacerbates charge accumulation on the epoxy resin surface, further intensifying the surrounding electric field distortion. When the electric field distortion surpasses the local breakdown field strength of the gas between two particles, local discharge occurs, evolving into a flashover phenomenon along the epoxy resin surface.

On a more microscopic level, considering energy band structure and charge dissipation, external excitation and electric field distortion lead to the release of electrons from the valence band to the conduction band. Under the applied field strength, these liberated electrons initiate a flow of injection, generating new electrons through impacts on the surface. Metals, lacking a band gap, require less energy for electron jumps, making flash channel formation more likely. Due to external excitation reasons such as electric field distortion, thermal vibration occurs in the original thermal equilibrium state of the material, leading some electrons to break away from the conduction band of the metal dust. In the external field, the initial electrons hit resin surface, generating new electrons, and moving back and forth. The recombination forms a streamer, resulting in failure of the insulation between electrodes.

## 6. Conclusions

In this study, we constructed an experimental platform tailored for observing the adsorption-flashover of micron-nano dust, operational within GIS/GIL conditions. By doing so, we captured the motion behavior and adsorption process of micron-nano dust near epoxy resin insulators, shedding light on the unique physical phenomena during the adsorption process. The following conclusions were drawn:

(1) The adsorption states of micron-nano dust in the electric field encompass agglomerative adsorption along the insulator surface and diffusive adsorption along the ground electrode’s direction. Key factors influencing motion behavior include the initial position of micron-nano dust, its mass, material, and the externally applied voltage.
(2) Agglomerated adsorption of micron-nano dust leads to the formation of dust speckles on the epoxy resin insulator’s surface, playing a crucial role in inducing subsequent surface flashovers. As voltage increases, these dust spots undergo an “explosion”, generating an annular dust halo and other unique physical phenomena distinct from lifting adsorption. These phenomena are considered necessary conditions for inducing surface flashovers.
(3) Agglomeration adsorption of micron-nano dust speckles on the epoxy resin insulator’s surface creates a localized short-circuit region, reducing insulation distance between the high-voltage conductor and the grounding shell. This, combined with the drastic distortion of the gas-solid interface electric field induced by micron-nano dust speckles, intensifies local discharge between dust particles, making surface flashovers more likely. The presence of micron-nano dust resulted in a significant 71% reduction in the breakdown voltage of the test system.

## Figures and Tables

**Table 1:** Typical forces of dust.

Typical Force	Formula	Typical Force	Formula
Gravity	$G=\frac{4}{3}\pi a^{3}\rho_{\mathrm{dust}}g$	Van der Waals force	$F_{\mathrm{vdW}}=\frac{3}{2}\pi wa$
Buoyancy	$F_{b}=\frac{4}{3}\pi a^{3}\rho_{\mathrm{gas}}g$		$F=\frac{2\pi wa}{3}\left[\frac{1}{4}{\left(\frac{h}{z_{0}}\right)}^{-8}-{\left(\frac{h}{z_{0}}\right)}^{-2}\right]$
Electric force	$F_{E}=qE=q\frac{U_{\mathrm{dc}}}{r\ln\frac{R_{2}}{R_{1}}}$		$F_{\mathrm{Rumpf}}=\frac{A}{6}\left[\frac{r_{0}a}{z_{0}^{2}\left(a+r_{0}\right)}+\frac{a}{{\left(z_{0}+r_{0}\right)}^{2}}\right]$
Coulomb force	$F_{q}=\frac{1}{4\pi \varepsilon_{0}}\frac{q_{1}q_{2}}{{\left(2a+h\right)}^{2}}$	Adhesive force	$F_{\mathrm{adhe}}=w\frac{\pi }{180}a\arccos(1-\frac{4}{3}\pi a^{3}\rho_{\mathrm{Al}}gk_{\mathrm{tension}})$

## Data Availability

Data are contained within the article.

## References

[1] Hu Q., Li Q., Liu Z., Xue N., Ren H., Haddad M. (2022). Surface Flashover Induced by Metal Contaminants Adhered to Tri-Post Epoxy Insulators under Superimposed Direct and Lightning Impulse Voltages. Polymers.

[2] Zhou H., Ma G., Wang C., Wang J., Zhang G., Tu Y., Li C. (2021). Review of charge accumulation on spacers of gas insulated equipment at DC stress. CSEE J. Power Energy Syst..

[3] Magier T., Tenzer M., Koch H. (2018). Direct current gas-insulated transmission lines. IEEE Trans. Power Deliv..

[4] Kumada A., Okabe S. (2004). Charge distribution measurement on a truncated cone spacer under DC voltage. IEEE Trans. Dielectr. Electr. Insul..

[5] Hasegawa T., Yamaji K., Hatano M., Aoyagi H., Taniguchi Y., Kobayashi A. (1996). DC dielectric characteristics and conception of insulation design for DC GIS. IEEE Trans. Power Deliv..

[6] Xue N., Yang J., Shen D., Xu P., Yang K., Zhuo Z., Zhang L., Zhang J. (2019). The Location of Partial Discharge Sources inside Power Transformers Based on TDOA Database with UHF Sensors. IEEE Access.

[7] Zhou H., Ma G., Li C., Shi C., Qin S. (2017). Impact of temperature on surface charges accumulation on insulators in SF6 filled DC-GIL. IEEE Trans. Dielectri CS Electr. Insul..

[8] Qi B., Li C., Hao Z., Geng B., Xu D., Liu S., Deng C. (2011). Surface discharge initiated by immobilized metallic particles attached to gas insulated substation insulators: Process and features. IEEE Trans. Dielectr. Electr. Insul..

[9] Wang J., Wang J., Hu Q., Chang Y., Liu H., Liang R. (2020). Mechanism analysis of particle-triggered flashover in different gas dielectrics under DC superposition lightning impulse voltage. IEEE Access.

[10] Ma G., Zhou H., Wang Y., Zhang H., Lu S., Tu Y., Wang J., Li C. (2020). Flashover behavior of cone-type spacers with inhomogeneous temperature distribution in SF_6_/N_2_-filled DC-GIL under lightning impulse with DC voltage superimposed. CSEE J. Power Energy Syst..

[11] Chang Y., Liu Z., Li Q., Xue N., Wang J., Manu H. (2022). Capture mechanism and optimal design of microparticle traps in HVAC/HVDC gas insulated equipment. IEEE Trans. Power Deliv..

[12] Liang R., Hu Q., Liu H., Wang J., Li Q. (2020). Research on Discharge Phenomenon Caused by Cross-Adsorption of Linear Insulating Fiber and Metal Dust under DC Voltage. High Voltag..

[13] Wang J., Li Q., Gong Y., Su B., Li Z., Liu H., Wang J., Ren H. (2022). Enhancement of surface electrical performances of epoxy film by blending with fluorinated and crosslinking monomer. Mater. Lett..

[14] Zhang L., Lu S., Li C., Cui B., Wu Y. (2020). Movement and Discharge Characteristics of Micron-Scale Metal Dust in Gas Insulated Switchgear. Trans. China Electrotech. Soc..

[15] Li X., Liu W., Xu Y., Cheng W., Bi J. (2020). Surface charge accumulation and pre-flashover characteristics induced by metal particle on 1100kV GIL post insulator surface under AC voltage. High Volt..

